# Leaf–Root–Soil Stoichiometric Characteristics in Different Shrub Ages of *Ammopiptanthus mongolicus* 

**DOI:** 10.3390/plants12173103

**Published:** 2023-08-29

**Authors:** Xue Dong, Dehao Xu, Danyang Wang, Chunxia Han, Yaru Huang, Jingbo Zhang

**Affiliations:** 1 Experimental Center of Desert Forestry, Chinese Academy of Forestry, National Long-Term Scientific Research Base of Comprehensive Control in Ulan Buh Desert, Inner Mongolia Dengkou Desert Ecosystem National Observation Research Station, Dengkou 015200, China; dongxue98765@126.com (X.D.); dan_yang_wang@163.com (D.W.); hanchunxia0116@126.com (C.H.); hu_angyaru@126.com (Y.H.); 2 Institute of Desertification Studies, Chinese Academy of Forestry, Beijing 100091, China; 3 Inner Mongolia Yellow River Project Management Center, Dengkou 015200, China; xudehao@126.com

**Keywords:** *Ammopiptanthus mongolicus*, ecological stoichiometry, different stand age, leaf-root-soil, nutrient balance

## Abstract

The ecological indicators for the growth and restoration of *A. mongolicus* populations are important for grasping the regulatory mechanisms of the biogeochemistry cycle, and for providing basic data for the prediction and evaluation of the evolution characteristics of natural *A. mongolicus* populations. We conducted studies on the eco-stoichiometric characteristics of natural *A. mongolicus* in different shrub ages, in order to understand the nutrient limitations for the growth and development of *A. mongolicus* and the synergy between the soil, leaves and roots, and to explore the C, N and P stoichiometric characteristics on *A. mongolicus*. The results showed the following: (1) The response of C, N and P stoichiometric characteristics in the leaves, roots and soil to changes in shrub age was not completely consistent. The leaf C content was young shrub> mature shrub> middle age shrub. The C content in the root system and C and N content in the soil showed an upward trend with increasing shrub age. The N and P contents of the root system and the P content of the soil showed a downward trend with increasing shrub age. The stoichiometric ratios C:N, C:P and N:P in the leaves, roots and soil showed an upward trend, and the N:P ratios in the leaves and roots were similar. (2) Among the stoichiometric characteristics of the leaves, C, N and P, leaves P and C:P are the most sensitive to shrub age changes, and have ecological implications for the growth and population dynamics of *A. mongolicus*. The average N:P ratios of young *A. mongolicus* leaves in young, middle-aged and mature shrubs were 15.32, 18.23 and 21.76, respectively. It can be seen that with an increase in shrub age, the growth of *A. mongolicus* gradually shifted from being jointly restricted by N and P to being more restricted by P. (3) The N content and the C∶N and N∶P ratios of *A. mongolicus* are classified as “strictly homoeostasis “, which shows strong plant homoeostasis for environmental adaptability. The N supplemented by symbiotic nitrogen fixation makes *A. mongolicus* have strong N internal homoeostasis. Therefore, in a desert grassland with low N content, the growth process of *A. mongolicus* may be easily restricted by P due to the additional N absorbed by it. (4) The C, N and P contents of the leaves, roots and soils of the three shrubs were shown as leaf > root > soil, and the difference was significant (*p* < 0.05). The correlation analysis showed that the C, N and P contents of the soil, roots and leaves and their stoichiometric ratio characteristics of the three shrubs showed a certain correlation. Among them, the P content of the soil was significantly related to the N:P ratio of the leaves and roots. Therefore, P is likely to become a limiting factor in the plant growth and repair process of the plant ecosystem in the *A. mongolicus* population. In summary, during the growth of *A. mongolicus*, special attention should be paid to the balance of nutrients. In order to improve its productivity, it is recommended to reasonably apply P fertilizers in the process of tending management to enhance the soil nutrient status and improve plant nutrient utilization efficiency and homoeostasis.

## 1. Introduction

C:N:P stoichiometry is an important tool for exploring and predicting ecological processes, functions, the dynamic balance and the interactions of various elements [1]. As important biogenic elements, C, N, P and their ratios are effective ways to study plant growth and development, as well as the exchange of substances and energy in ecosystems, which indicate the limits of nutrients and the decomposition of organic matter [2,3]. The leaf is the main organ for photosynthesis in plants, and the feedback between leaves and the C, N and P elements in soil is particularly close. Some studies have shown that the nutrient content in the leaf may better reflect the ability of the soil nutrient supply [4,5]. In addition, soil is the main source of nutrients for plant growth, which plays an important role in its regulation. Therefore, the plant–soil feedback mechanism is helpful to explain the nutrient relationship between plant leaves and soil [6]. The plant root system is also the main organ to absorb nutrients and water [7,8], which is the link between the plant and soil. Clearly, there is an urgent need for a comprehensive study about the stoichiometric characteristics of the C, N and P of roots, leaves and soil. With the growth of plants, the composition of the community ecosystem, the soil properties and the internal environment will also change, which will affect the distribution pattern of nutrients in the plant–soil system, in studying the variations in leaf–root–soil stoichiometric characteristics with forest age. It is helpful to systematically reveal the characteristics of plant–soil nutrient distribution at different growth stages.

As China’s first batch of rare and endangered relics under special protection, *Ammopiptanthus mongolicus* has special taxonomic status and extremely high scientific research value. In addition, it is also the only evergreen broad-leaved shrub plant in the arid region of northwest China [9], which has the characteristics of resistance to barren and wind erosion conditions. The shrub not only has a beautiful tree shape and bright colors, it also has economic potential in northern gardens and greening. In the case of reduced coverage due to the fall of other irrigation and grass plants, and the effectiveness of wind protection and sand fixation during the strong winds in winter and spring, it can still maintain its protective benefits. Due to the narrow distribution area, there are currently very few wild resources of *A. mongolicus*. Serious plant diseases, insect pests, the enlarged area of dry and senescent populations, and their poor self-repairing ability further endanger it. Previous studies have shown that forest age is one of the most important factors influencing the healthy growth of plants and the dynamic balance of soil nutrients. Forest age affects the metabolic capacity and element turnover rate in plants, resulting in differences in fertility status in rhizosphere soil at different forest ages [10]. In this experiment, young winter shrubs, middle-aged shrubs and mature shrubs were selected as the three stages for the growth of *A. mongolicus*. The C, N and P contents of the leaves, fine roots and soil, and the dynamic characteristics of the eco-stoichiometry were analyzed throughout their whole life. The contents of C, N and P and their ratios in the soil and plant leaves and fine roots were discussed, and correlations of stoichiometric characteristics among the leaves, fine roots and soils in various growth stages were analyzed. The leaf–root–soil nutrient feedback process helps to better understand the nutrient distribution pattern and biochemical cycle characteristics in different growth stages of *A. mongolicus*, which is important to effectively maintain the stability of natural *A. mongolicus* populations.

## 2. Results

### 2.1. C, N, P Contents and Stoichiometric Ratios of Leaves in Different Shrub Ages of A. mongolicus

**Figure 1:**
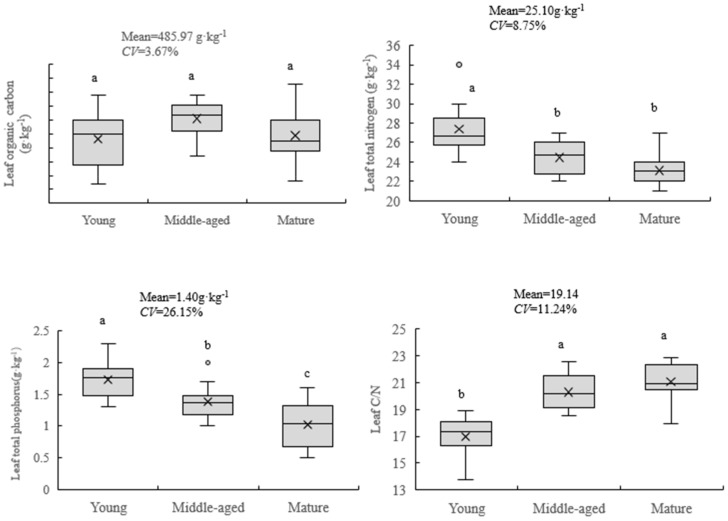

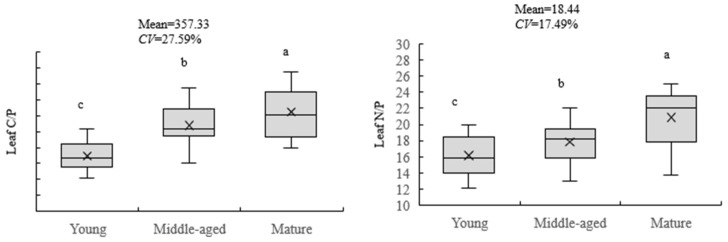
Effect on leaf stoichiometric characteristics of *A. mongolicus* with age. Note: Different small letters in the same column indicate significant differences at the *p* < 0.05 level.

It can be seen from Figure 1 that the range of organic C content in the leaves of *A. mongolicus* in different shrub ages ranges from 483.56 to 493.24 g·kg^−1^, and the average value (±standard deviation) is (485.97 ± 17.83) g kg^−1^. The minimum degree of leaf organic carbon content variation was only 3.67%. The range of N content in the leaves of *A. mongolicus* was 23.07~27.43 g·kg^−1^, the average value was (25.10 ± 2.20) g·kg^−1^, and the coefficient of variation was 8.75%. The change rule of the N content in the leaves of *A. mongolicus* at different shrub ages also showed a decreasing trend with the increase in shrub age and the young-aged shrubs, and the two reached a significant level (*p* < 0.01). The variation in P content in the leaves of *A. mongolicus* ranged from 1.06 to 1.79 g·kg^−1^, with an average value of (1.40 ± 0.37) g·kg^−1^. The variation degree of the P content in leaves was the largest, with a variation coefficient of 26.15%. The effects of shrub age on P in the leaves of *A. mongolicus* reached a significant level (*p* < 0.05), and showed a downward trend with the increase in shrub age. Generally speaking, during the process of growth of the *A. mongolicus* shrub, the overall change in leaf organic C content was not large, and the difference was not significant (*p* > 0.05); however, the difference in N and P content was significant (*p* < 0.05). The performance was middle-aged shrub> mature shrub> young shrub, while the leaf N and P contents were both young shrub > middle-age shrub > mature shrub.

The leaves’ C:N, C:P and N:P ratios of *A. mongolicus* showed an increasing trend with the increase in shrub age. The change range of C:N was 16.72~20.82, the average value was 19.14 ± 2.15. The change range of C:P was 256.18~453.13, and the average value was 357.33 ± 98.59. Since the difference in C content was not large, and the degree of variation in P content was large, the coefficient of variation of C:P reached a maximum of 27.59%, and the effect of shrub age on C:P of the leaves of *A. mongolicus* reached a significant level (*p* < 0.05). The variation range of N:P was 15.32~21.76, the average value was 18.44 ± 3.23, and the coefficient of variation was 17.49%. The N:P ratio of young *A. mongolicus* was 15.32, the N:P ratio was greater than 14 and less than 16, and the N:P difference was significantly different from that of the middle-aged and mature shrubs (*p* < 0.05), and that of N in the middle-aged and mature shrubs. The N:P ratios were 18.23 and 21.76, respectively, and both were greater than 16. It can be seen from this that the growth of *A. mongolicus* in young age is mainly limited by N and phosphorus. As the age of the shrub increases, the growth of *A. mongolicus* in the middle-aged and mature shrubs is mainly restricted by phosphorus.

### 2.2. C, N, P Contents and Stoichiometric Ratios of Root in Different Shrub Ages of A. mongolicus

**Figure 2:**
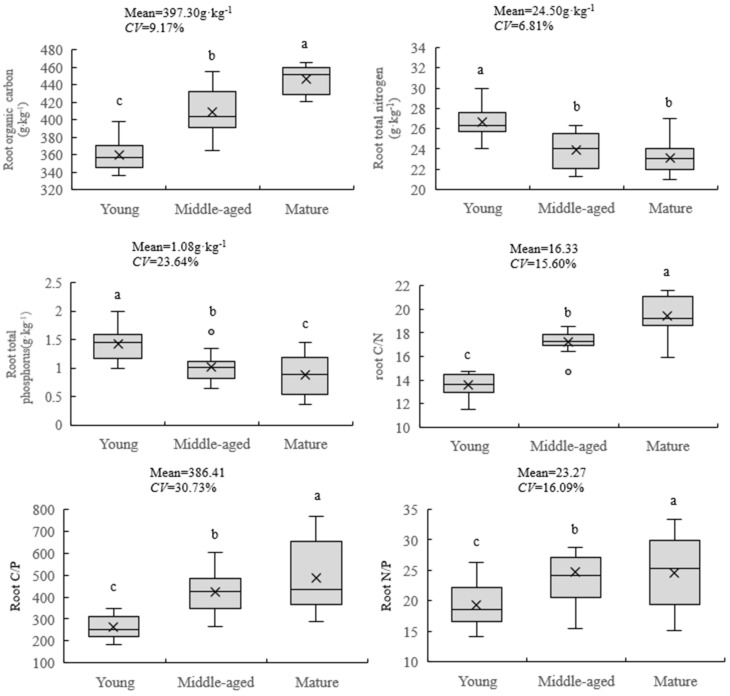
Effect on root stoichiometric characteristics of *A. mongolicus* with age. Note: Different small letters in the same column indicate significant differences at the *p* < 0.05 level.

It can be seen from Figure 2 that the organic C content in the root system of *A. mongolicus* at different ages varies from 358.26 to 430.38 g·kg^−1^, with an average value (± standard deviation) of (397.30 ± 36.43) g·kg^−1^. The change rule of root C content in *A. mongolicus* at different shrub ages showed an increasing trend with the increase in shrub age. The coefficient of variation was 9.17%. The root N content of *A. mongolicus* varied from 23.07 to 26.33 g·kg^−1^, with an average value of (24.50 ± 1.67) g·kg^−1^, and the minimum variation of total N content in the root system was only 6.81%. The change rule of root N content in *A. mongolicus* at different shrub ages showed a decreasing trend with the increase in shrub age, and young shrubs and both reached significant levels. (*p* < 0.01). The variation range of P content in the root system of *A. mongolicus* was 0.86~1.36 g·kg^−1^, and the average value was (1.08 ± 0.26) g·kg^−1^. The variation degree of P content in the root system was the largest with a coefficient of variation of 23.64%. The influence of shrub age on the root P of *A. mongolicus* reached a significant level (*p* < 0.05), and it showed a downward trend with the increase in shrub age. Overall, shrub age has a significant effect on the root C, N and P content and the stoichiometric ratios of *A. mongolicus* (*p* < 0.05). The roots’ C:N, C:P and N:P ratios of *A. mongolicus* showed an increasing trend with the increase in shrub age. The change ranges of C:N and N:P were 13.61~18.66 and 19.36~26.83, the average values were 16.33 ± 2.55 and 23.27 ± 3.74, and the coefficients of variation were 15.60% and 16.09%, respectively. The C:P ratio varied from 263.43 to 500.44, with an average value of 386.41 ± 118.76. Since the difference in P content is not large, and the variation degree of C content is large, and the change trend of the two is opposite; the coefficient of variation of C:P reached a maximum of 30.73%, and the effect of shrub age on the root C:P of *A. mongolicus* reached very significant level (*p* < 0.01).

### 2.3. C, N, P Content Changes and Stoichiometric Ratios of Soil under A. mongolicus Shrub in Different Ages

**Figure 3:**
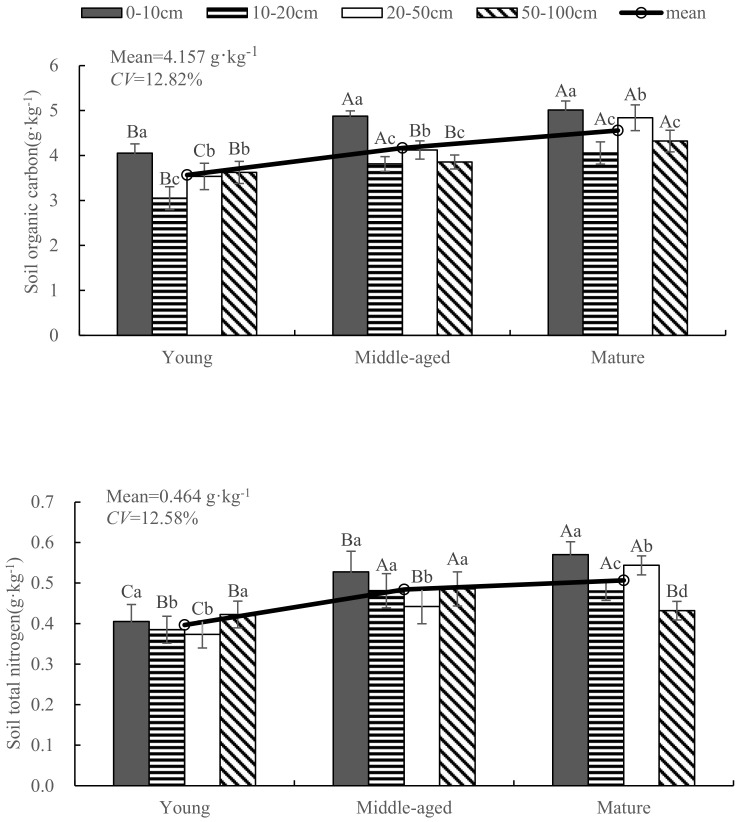

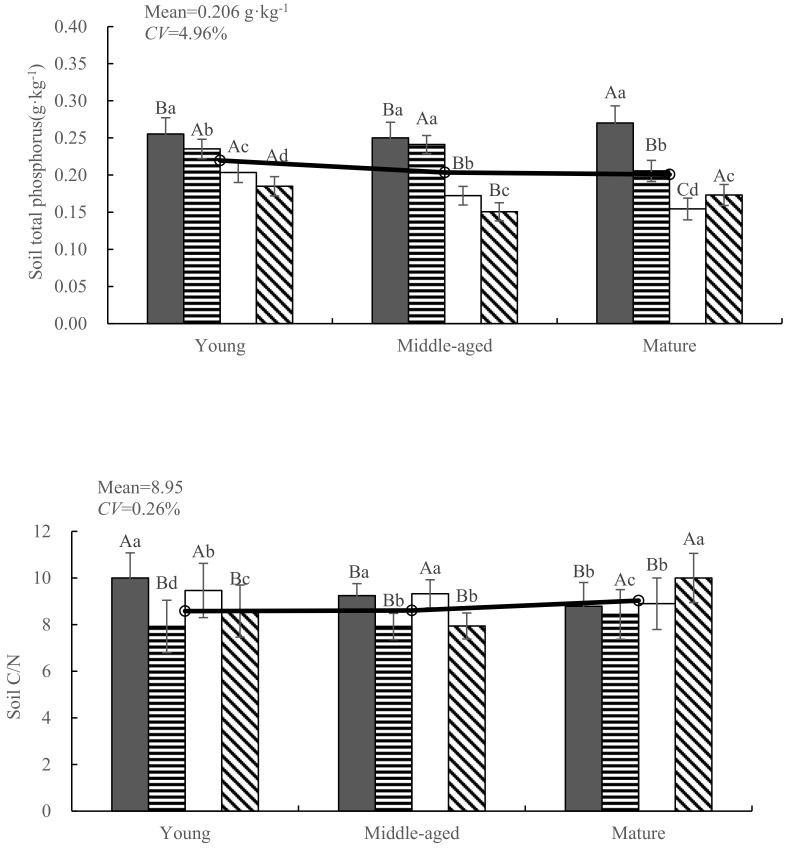

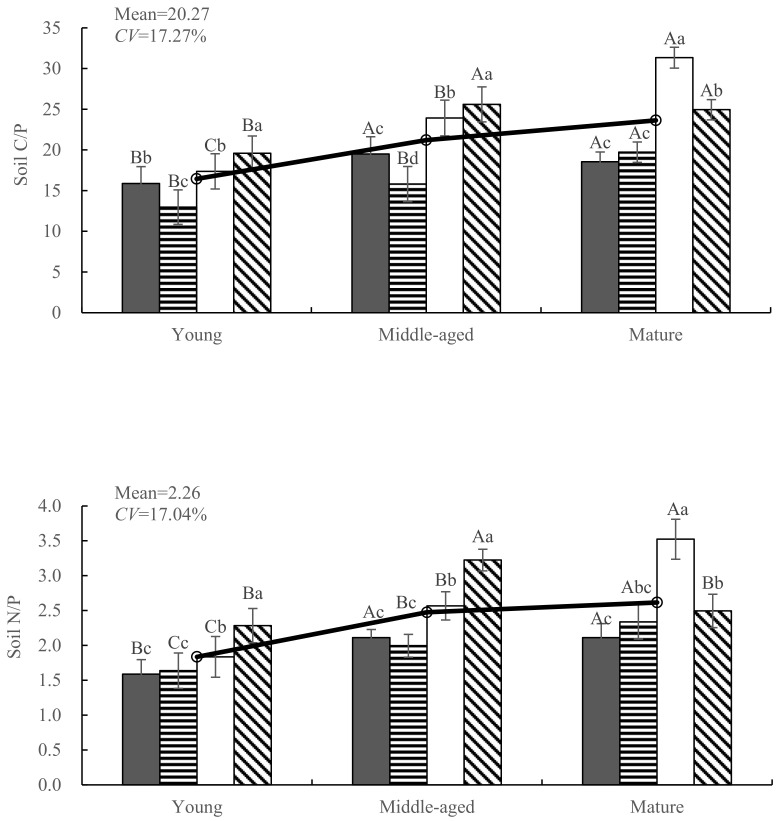
Effect on soil stoichiometric characteristics of *A. mongolicus* with age. Note: Upper case letters represent significant differences in different ages in the same soil layer (*p* < 0.05). Lower case letters represent the significant differences in different soil layers in the same age (*p* < 0.05).

It can be seen from Figure 3 that the range in C content of soil under *A. mongolicus* shrub is 3.55~4.56 g·kg^−1^, and the average value (± standard deviation) is (4.16 ± 0.53) g·kg^−1^. The soil organic carbon content of *A. mongolicus* in different shrub ages was significantly different (*p* < 0.05), and the degree of organic carbon content variation was 12.82%. The content of soil organic carbon and total N in *A. mongolicus* showed an upward trend with the increase in shrub age, but the content of total P showed a downward trend. The range of N content of soil under the shrubs ranged from 0.398 to 0.508 g·kg^−1^, the average value was (0.464 ± 0.058) g·kg^−1^, and the coefficient of variation was 12.58%. The soil N content in the young shrubs of *A. mongolicus* was significantly different from those in the middle-aged and mature shrubs (*p* < 0.01). The minimum variation degree in total P content in the soil was only 4.96%. The contents of C, N and P in the surface layer (0–10 cm) of each shrub age were significantly higher than those in other soil layers, and the differences among different soil layers of the same shrub age were significant. The contents of C, N and P and their stoichiometric ratios in the soil under shrubs fluctuated with the depth of the soil layer, but there was no obvious change rule. The soil C:N, C:P and N:P ratios under the *A. mongolicus* shrubs increased with the age of the shrub, and the change law was the same as for the leaves and roots; the average values were 8.95 ± 0.02, 20.27 ± 3.50 and 2.26 ± 0.39, respectively. The coefficients of variation were 0.26%, 17.27% and 17.04%, respectively. The C:N value of the soils of *A. mongolicus* in different shrub ages was relatively stable, while the soil C:P had significant differences among the shrub stands (*p* < 0.05). The soil N:P ratio in young shrubs was significantly different from that in the middle-aged shrubs and mature shrubs (*p* < 0.01).

### 2.4. Correlations between C, N, P Contents and Stoichiometric Ratios in Leaves, Roots and Soil of A. mongolicus

Although different organs of plants have different functions, only through the synergy of different organs can plants complete a series of complex physiological and biochemical activities. Therefore, there must be some connections between different organs. In the analysis of leaf and root element content data, the root C element content was negatively correlated with the leaf N and P element contents, but positively correlated with the leaf stoichiometric ratio; the root N and P was positively correlated with the leaf N and P element contents, but there was a negative correlation between the stoichiometric ratio of the leaves. In general, the positive correlations were more than the negative correlations. There was a significant correlation found between the content of C in the root and leaf N and C:N (*p* < 0.05). There was a very significant positive correlation between the content of P in the root and the contents of N and P in the leaf (*p* < 0.01). Soil is one of the main sources of root nutrition. All physiological and biochemical processes of the root system are completed in the soil. Therefore, the content of soil elements has an important effect on the root system of plants. The ratios of root C:P and N:P were greatly affected by the contents of C, N and P in the corresponding soil (*p* < 0.05). The correlation analysis of stoichiometric characteristics of leaves and soil C, N and P in different shrub ages showed that soil C and leaf C were positively correlated, soil N and leaf N were negatively correlated, but none reached significant levels; meanwhile, the soil P and leaf P showed a significant positive correlation (*p* < 0.05). The soil C was significantly negatively correlated with soil P and leaf P (*p* < 0.05), but extremely and significantly positively correlated with the soil C:P ratio (*p* < 0.01), and significantly and positively correlated with the soil N:P and leaf C:N (*p* < 0.05). The soil N was significantly and positively correlated with the soil N:P ratio (*p* < 0.05), and extremely and significantly positively correlated with the leaf N:P ratio (*p* < 0.01). The soil P was significantly and positively correlated with the leaf N and P (*p* < 0.05), but significantly and negatively correlated with the soil C and leaf C:N ratio (*p* < 0.05), and with the soil N:P and soil C:P ratios in a significant negative correlation (*p* < 0.01). The soil C:P and soil N:P ratios were significantly positively correlated (*p* < 0.05). It can be seen that the functional traits of the leaves and roots of *A. mongolicus* are jointly affected by the soil’s organic carbon and total phosphorus content (Table 1).

### 2.5. Stoichiometric Homoeostasis of A. mongolicus

**Figure 4:**
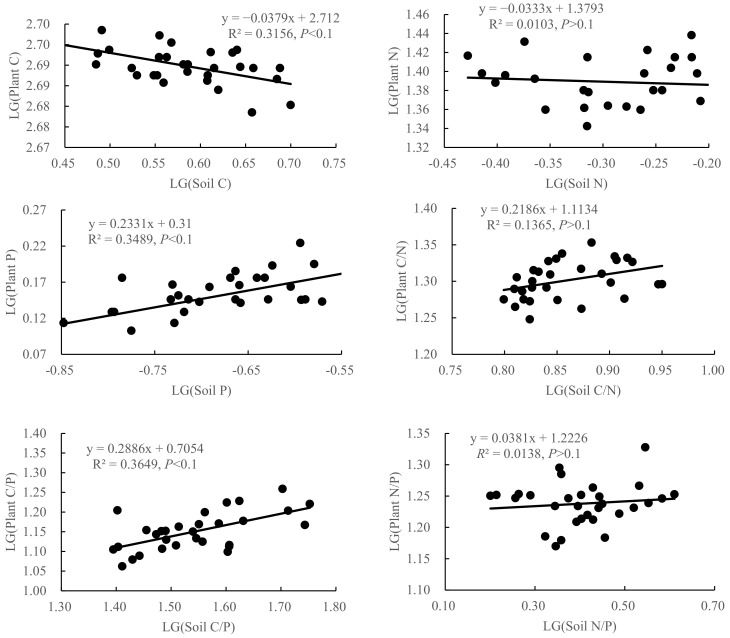
Relationships between log10-transformed C, N and P contents and stoichiometric ratios in plants and soil of *A. mongolicus*.

The stoichiometric homoeostasis analysis results of the C, N and P contents and their stoichiometric ratios in plants and soil are shown in Figure 4. The N content of *A. mongolicus* and the ratio of C/N to N/P were classified as “strict homoeostasis” (*p* > 0.1). The C content of *A. mongolicus* 1/H_c_ = 0.038 (*p* < 0.1) and the P content 1/H_p_ = 0.233 (*p* < 0.1) both belonged to “steady state”, and the C/P ratio 1/H_c/p_ = 0.289 (*p* < 0.1) belonged to “weak steady state”, indicating that the nitrogen fixation characteristics of *A. mongolicus* made it obtain sufficient N in the N-deficient desert grassland, and its plant N content was less affected by changes in the soil nutrient contents, which was relatively stable. Although the P content of *A. mongolicus* was greatly affected by the soil nutrient content, its N:P ratio was basically not affected by the soil P content, showing strong homoeostasis.

## 3. Discussion

### 3.1. The Stoichiometric Characteristics of Carbon, Nitrogen and Phosphorus in the Natural A. mongolicus Shrub

In this study, the average carbon content of the leaves in the natural *A. mongolicus* shrub is 484.38 g·kg^−1^, which is higher than that of the 492 terrestrial plants (464.0 g·kg^−1^) and of arid area plants (338.0 g·kg^−1^) [11,12]. It shows that the content of organic matter in the leaves of *A. mongolicus* is high. It can produce enough organic matter to allocate to the leaves through photosynthesis in different shrub ages, the structural carbon content in the leaves of *A. mongolicus* is very high, and the variation is relatively small and relatively stable. This is because the carbon in plants generally does not directly participate in plant production activities, but mainly plays a role in the skeletons of plants. The leaf carbon content is relatively higher in middle-aged shrubs because the shrubs grow rapidly at a young age and the metabolic activity is strong, and enzymes are needed to synthesize a large amount of protein. However, the accumulation of carbon-rich structural materials led to an increase in the carbon content in middle-aged shrubs and mature shrubs [13]. The nitrogen and phosphorus contents of the leaves of *A. mongolicus* are 26.80 and 1.60 g·kg^−1^, respectively, which are significantly higher than the global leaf N content (20.62 g·kg^−1^, 20.09 g·kg^−1^) [11,14] and the arid regions leaf N content (18.1 g·kg^−1^) [12]; the phosphorus content is much lower than the global average phosphorus content of plant leaves (1.99 g·kg^−1^, 1.77 g·kg^−1^) [11,14], but it is significantly higher than that in our region (1.3 g·kg^−1^) [15,16,17]. The nitrogen and phosphorus decrease with shrub age, but the N:P ratio increases. It indicates that the growth rate of the mature shrub of *A. mongolicus* is relatively slow. However, the larger the individual plant is, the higher the nutrient content required for survival and growth. With the increase in shrub age, the P content in the leaves of *A. mongolicus* decreased significantly, which may be one of the important reasons for the aging and death of *A. mongolicus*.

The C:N, C:P and N:P ratios showed an increasing trend with the age of the shrub, which can be an excellent indicator for the growth of *A. mongolicus* in different periods. Both the leaf C:N and C:P ratios represent the ability of plants to assimilate carbon, reflecting their utilization efficiency of plant nutrients. Generally, plants with lower leaf C:N and C:P ratios have faster growth rates [2]. In this study, the average of the C:N and C:P ratios in the growth process of *A. mongolicus* gradually increased from the young shrub to the mature shrub, which showed that the growth rate of *A. mongolicus* in the nutrient-poor habitat slowed down with the increase in stand age. Plants grow fast at a young age, so the leaf C:N and C:P ratios are the smallest. Then, the growth rate is relatively slow as the shrub gradually grows into middle-aged and mature individuals, and the leaf C:N gradually stabilizes. Studies have shown that the leaf N:P ratio can indicate plant nitrogen limitation or phosphorus limitation. Simply, an N:P value less than 14 usually means nitrogen limitation, while an N:P value greater than 16 means plants are more restricted by phosphorus and, between the two means, that plants are restricted by both nitrogen and phosphorus [18,19,20]. The average of N:P in the leaves of *A. mongolicus* was 18.44, indicating that the growth was more restricted by phosphorus. If *A. mongolicus* is classified by shrub age, the average N:P value of a young shrub is 15.32, which is limited by N and P. The average N:P values of a middle-aged shrub and mature shrub are 18.23 and 21.76, respectively, which are limited by P. Therefore, the growth of *A. mongolicus* is gradually restricted from both N and P to be more restricted by P with shrub age. This is mainly because as the growth rate of the aboveground part slows down, as a legume, the plant has rhizobia in the root system, which can fix nitrogen itself to meet the plant’s growth needs.

The root C, N and P contents showed a regular distribution trend at different shrub ages. With an increase in the age of the shrub, the C content showed a significantly and gradually increasing trend, indicating that the carbon fixation ability in the root system of *A. mongolicus* becomes stronger and stronger. The N and P contents showed a decreasing trend with increasing shrub age, which may be due to larger individuals of *A. mongolicus* needing more nutrients, and the root N and P contents being in short supply. In addition, the root C:N, C:P and N:P ratios showed an increasing trend with shrub age, which may be due to the decrease in the N and P contents in the root system. On the other hand, with the increase in shrub age, the content of C in the soil also increased because the litter also increased. In this study, the average N and P contents of the roots of *A. mongolicus* were (24.50 ± 1.67) and (1.08 ± 0.26) g·kg^−1^, respectively, which were higher than the average level of 9.2, 1.0 g·kg^−1^ of terrestrial plants in China [21,22,23,24]. The average N:P ratio of the *A. mongolicus* root system is 23.27, which is higher than China’s average of 14.27. The “growth rate hypothesis” [2] indicates that a large amount of P would be allocated to rRNA during the period of rapid growth, and ribosomes can quickly synthesize a large number of proteins, thereby showing low N:P ratios. The N:P ratio of the root system increased significantly with shrub age, indicating that the growth rate of the roots decreased with the age of *A. mongolicus*. At each growth stage of *A. mongolicus*, the C, N and P contents of each component were expressed as leaf> root system> soil. This is because the leaf is the main place for plants to use nutrient elements. The roots transport most of the C, N and P elements absorbed from the soil to the leaves for their absorption and synthesis of organic matter, while the leaves and roots return part of the nutrient elements to the soil in the form of litter to be mineralized and decomposed through microbial decomposition, resulting in less nutrient elements finally entering the soil [25,26,27,28].

### 3.2. Relationships between Soil and Plant Stoichiometric Characteristics of A. mongolicus

The nutrient elements required for plant growth are mainly derived from C, N and P in the soil, which are essential nutrient elements that affect the growth and development of plants, and directly determine the level of plant community productivity in the process of plant growth and development [29,30,31,32,33]. The content of C in the leaves and roots of *A. mongolicus* was significantly higher than that in the soil, which indicated that the effect of C in the soil had little effect on *A. mongolicus*, which may be due to the main source of photosynthesis. For the root system, there is a strong correlation between the root system and the soil elements for the content and ratio of each element, which showed that shifts in soil elements have a greater impact on each element of the root system, and because all physiological and biochemical processes of the root system are completed in the soil. On the whole, there are more positive correlations than negative correlations between each element of the soil and each element of the plant root, which indicate that an increase or decrease in the element in the soil will promote or inhibit the increase in some elements in the roots to a certain extent. The soil and root systems play different roles in the nutrient cycle, so there must be some connection between them. We found that there is a significant and positive correlation between the soil N content and the root N:P ratio of *A. mongolicus*, while the soil P content and the root N:P ratio have a significant and negative correlation. This phenomenon shows that soil drives the nutrient uptake capacity of plants, and verifies the limitations of phosphorus on plant growth in this region. The root system has the functions of fixing plants, absorbing water required for plant growth and development, fixing inorganic nutrients and a small amount of organic nutrients, and synthesizing growth-regulating substances. The leaf is the main place for photosynthesis and respiration, and has the function of storing nutrients. The main source of nutrients is air, and some are absorbed from the root system, so there is a certain degree of relationship between the leaf and root elements.

### 3.3. Stoichiometric Homoeostasis in Plant and Soil of A. mongolicus

The stoichiometric homoeostasis of plants is positively related to population stability, and plants with higher homoeostasis have a stronger ability to use nutrients and adapt to the environment [34]. As an evergreen shrub in desert areas, *A. mongolicus* has strong stability in coping with environmental changes through its own physiological and biochemical adjustment mechanisms. In this study, the N content, C∶N and N∶P ratios and stoichiometric homoeostasis of *A. mongolicus* is classified as “strict homoeostasis “, C and P are classified as “steady state”, and the C/P ratio is classified as “weak steady state”. The reasons may be as follows: (1) *A. mongolicus* has strong nutrient regulatory ability, which may be the result of its adaptation to barren environments through a long-term evolution process. (2) During its growth, *A. mongolicus* relies on a large amount of C to carry out the metabolism and synthesis of organic substances, thus it can resist environmental stress and pests, and the change in the soil C/P ratio with shrub age may lead to a decrease in plant homoeostasis. (3) The increase in N concentration in plant tissues is generally accompanied by an increase in P concentration. The higher N:P homoeostasis in plant means that a change in N:P of *A. mongolicus* is smaller than that of N and P, respectively. The changes in N and P absorption during its growth are more consistent, indicating that *A. mongolicus* has a conservative nutrient utilization strategy [35]. Yu Qiang [36] pointed out that high homoeostasis and a conservative nutrient utilization strategy are the keys to the survival of plants during drought and barren conditions. Therefore, the stoichiometric homoeostasis depends on the biological characteristics of plants. Plants have stoichiometric homoeostasis for chemical nutrients that are diverse from each other.

## 4. Materials and Methods

### 4.1. Research Site

**Figure 5:**
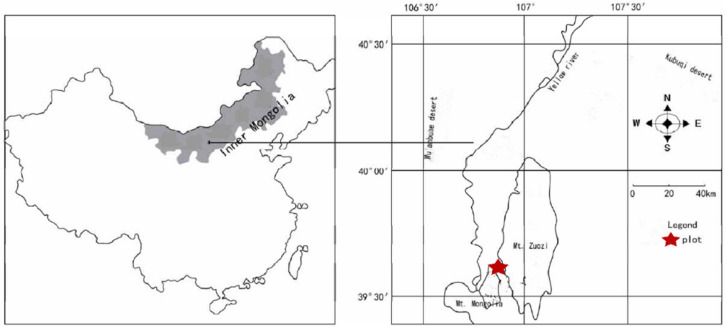
Schematic diagram of the geographical location of the study area.

The research area is a grassland to desert ecosystem vegetation zone, spanning the Etuoke Banner and Wuhai City in the west of the Ordos Plateau (Figure 5). The area is centered on the triangular core area composed of Helan Mountain–Albas Mountain–Wolf Mountain. The geographical coordinates are 106°40′–107°44′ E, 39°14′–40°11′ N, and the total area is 555,849 hm^2^. The study area is characterized by a continental climate, with high wind and sand, dry climate, strong solar radiation, and large temperature differences between winter and summer. The annual average temperature is 9.6 °C. The hottest month is July, with extreme maximum temperatures up to 39 °C, and the coldest month is January, with extreme minimum temperatures as low as −35 °C. The frost-free period is 129 days. The average annual precipitation is 272.3 mm, and the distribution is uneven during the year, mostly concentrated from June to August. The precipitation is 173.9 mm, accounting for 63.9% of the annual precipitation. The annual evaporation is 2470.4 mm, which is 9.1 times the precipitation, and the average annual wind speed is 3.4 m·s^−1^. The zonal soil in the protected area is sandy gravel desert calcareous soil; the soil is barren and strongly alkaline, with a pH value of 9.0–10.0. The community composition includes *Zygophyllum xanthoxylum*, *Tetraena mongolica*, *Helianthemum songaricum*, *Potaninia mongolica*, *Reaumuria songarica* and other plants.

### 4.2. Sample Selection and Setting

In the same habitat, the plot conditions are similar. Within the natural *A. mongolicus* community with shrubs of different ages intermingled with each other, the growth indicators of *A. mongolicus* were investigated during the period when the plants had the largest biomass in early August 2017 (Table 2), based on the detection of each shrub in the study area, taking the height of the cluster (H, cm), ground diameter (D, mm) and the crown width (C, m^2^) as the index parameters. The natural *A. mongolicus* was divided into three different age classes: young shrubs, middle-aged shrubs and mature shrubs. The stands were adjacent to each other, and the plot area was 1 km^2^. Five 100 × 100 m quadrats were randomly set up in the plot, and shrubs that were close to the growth indicators of each shrub age and had good growth were found in the set plots, and then marked as the sample shrubs. For sampling, 20 shrubs of each type of stand were selected.

### 4.3. Sample Collection and Determination Method

There was no rainfall within one week before sampling, and the weather was clear. In each shrub group, 20 plants with better growth were randomly selected; each shrub was selected from the leaves that were fully extended, mature and free from diseases and insect pests on the branches. Then, at least 200 g of the harvested leaves were mixed and installed into a kraft paper bag. A 1.5 m deep trench was dug to take the main roots and lateral roots (Wilcox, et al., 2004), peeling off the impurities and mixing them; at least 100 g of the root samples were kept in sealed storage. We selected 0–100 cm surface soil samples in four layers: 0–10, 10–20, 20–50 and 50–100 layers under the crown of *A. mongolicus* (east, west, south, north), and mixed them evenly, putting them into aluminum boxes and marking them. We took 3 repeats under each shrub and 3 shrub groups. There were 20 shrubs in each shrub stand, and a total of 720 soil samples were taken. When collecting the soil samples, falling objects on the surface were removed, and the soil was collected at a distance of 5 cm from the root of the shrub. Then, all of the collected samples were taken back to the laboratory, with the leaves and roots at 105 °C for 30 min, dried at 75 °C for 48 h to a constant weight, and the dried leaves and roots were put through a fine sieve, and a quartile method was used for sampling. For the measurement of organic C, total N and total P content, the soil sample in the aluminum box was taken out and placed on a plastic film to air-dry, and the dry weight was measured. Then, the grass roots and stones were removed, ground, and passed through a 0.15 mm sieve. The potassium dichromate external heating method was used to determine the content of organic carbon in the leaves, roots and soil; after digestion via the H_2_SO_4_–H_2_O_2_ method, the total N content in the leaves, roots and soil was determined with the Kjeldahl method. The molybdenum antimony colorimetric method was used to determine the total phosphorus content in the leaves, roots and soil.

Coefficient of variation% (CV) = standard deviation of traits/average of traits × 100%

### 4.4. Data Analysis

With reference to the method of Bai et al. [37], a continuously variable regulation parameter (H) was introduced to quantify the stoichiometric homoeostasis of “leaf soil” in the ecosystem. The method is as follows: first, convert the C, N and P contents and the stoichiometric data of the leaves and soil with 10 as the bottom, then conduct regression analysis on C (1/H_C_), N (1/H_N_), P (1/H_P_), C/N (1/H_C/N_), C/P (1/H_C/P_) and N/P (1/H_N/P_) between the leaves and soil, and conduct a unilateral difference significance test (α = 0.1). If the regression relationship is not significant (*p* > 0.1), then 1/H = 0, and the system is considered to be in “strict homoeostasis”. A system with 1/H = 1 is not considered stable. If the data are significant (*p* < 0.1) and the regression coefficient is 0 < 1/H < 1, they are classified as 0 < 1/H < 0.25, belonging to “steady state”, 0.25 < 1/H < 0.5 belonging to “weak steady state”, 0.5 < 1/H < 0.75 belonging to “weak variability”, and 1/H > 0.75 belonging to “strong variability”. One-way ANOVA was used to test whether the C, N and P contents and stoichiometric ratios of the leaves, roots and soils of *A. mongolicus* were significantly different among the different shrub ages; then, correlation analysis was used to explore the *A. mongolicus*. The relationship between the C, N and P contents and stoichiometric ratios between the leaves, roots and soil of *A. mongolicus*. SPSS 19.0 (SPSS, Chicago, IL, USA) software was used for statistical analysis.

## 5. Conclusions

Through a systematic study on the carbon, nitrogen and phosphorus contents and stoichiometric ratios in leaf–root–soil of *A. mongolicus*, the mechanisms of nutrient ratio regulation and absorption efficiency were clarified. The stoichiometric characteristics of C, N and P in the leaves, roots and soil did not respond consistently to changing shrub age. The shrub age had a significant effect on the soil organic carbon and total N content under the *A. mongolicus* shrub (*p* < 0.05). With increasing shrub age, the contents of soil C and N showed an upward trend, and soil P showed a downward trend. There was no significant difference in the carbon content of leaves under different shrub ages (*p* > 0.05), but there was significant difference in the N and P contents (*p* < 0.05), and this decreased with increasing shrub age. The leaf P and C:P ratio are the most sensitive to changes in shrub age, and have an indicative significance for the growth and population dynamics of *A. mongolicus*. With increasing shrub age, the growth of *A. mongolicus* gradually shifts from N and P together to becoming more restricted by P. With increasing age, the root C content showed an increasing trend, while the root N and P contents showed a decreasing trend. There was an upward trend in the stoichiometric ratios of C:N, C:P and N:P of the leaves, roots and soil with increasing shrub age. Under the conditions of severe drought, *A. mongolicus* is restricted by water and nutrients. The nutrient content in plants reflects their adaptability to the environment. The soil nutrient conditions reflect the nutritional status of plants. Soil is the main source of plant nutrients. The C, N and P contents of the soil have certain correlations with the plant. At different growth stages, there are some differences in the absorption and utilization efficiency of soil nutrients by plants, which can adapt to water shortage and barren land by adjusting their physiological ecological modes. The soil nutrient content of *A. mongolicus* natural shrub is low, and the whole life growth process is mainly limited by P. Therefore, it is scientifically reasonable to increase P fertilizer appropriately during the growth and repair process of *A. mongolicus*.

## Figures and Tables

**Table 1:** Relationship between stoichiometric characteristics of *A. mongolicus* and soil.

Index	Leaf C	Leaf N	Leaf P	Leaf C:N	Leaf C:P	Leaf N:P	Soil C	Soil N	Soil P	Soil C:N	Soil C:P	Soil N:P
Root C	0.171	−0.494 *	−0.293	0.548 *	0.286	0.166	0.112	−0.256	−0.272	0.208	0.176	0.205
Root N	−0.214	0.396	0.294	−0.390	−0.279	−0.253	0.245	−0.185	−0.299	0.241	0.210	0.237
Root P	−0.233	0.894 **	0.892 **	−0.293	−0.273	−0.145	−0.359	0.428	0.315	−0.356	−0.354	−0.336
Root C:N	0.149	−0.397	−0.397	0.376	0.291	0.372	−0.370	0.288	0.256	−0.371	−0.110	−0.052
Root C:P	0.077	−0.397	−0.299	0.359	0.298	0.287	0.479 *	−0.489 *	−0.491 *	0.375	0.357	0.364
Root N:P	0.106	−0.399	−0.410	0.267	0.294	0.179	0.486 *	−0.492 *	−0.493 *	0.383	0.366	0.271
Soil C	0.371	−0.418	−0.487	0.516	0.237	0.169	1.000					
Soil N	0.348	−0.442	−0.439	0.338	0.274	0.609 **	0.398	1.000				
Soil P	−0.194	0.507 *	0.540 *	−0.485 *	−0.360	−0.760 **	−0.525 *	−0.271	1.000			
Soil C:N	0.251	0.339	0.354	−0.172	−0.150	−0.121	0.237	0.178	0.310	1.000		
Soil C:P	0.244	−0.532 *	−0.467 *	0.650 **	0.338	0.298	0.812 **	0.434	−0.891 **	−0.184	1.000	
Soil N:P	0.217	−0.378	−0.474 *	0.498 *	0.349	0.313	0.494 *	0.519 **	−0.409	−0.225	0.499 *	1.000

Note: *, *p* < 0.05; **, *p* < 0.01.

**Table 2:** Basic information about the shrub stand of *A. mongolicus* with different years after rejuvenation pruning and ages.

Shrub Stand	Height (cm)	Diameter (mm)	The Area of Crown (m^2^)	Branch Number
Young	25–40	4–7	0.5–2	5–15
Middle-aged	40–80	7–20	2–7.5	15–25
Mature	≥60	≥20	≥4.5	≥20

C: the area of crown (C = π × C_l_ × C_w_); C_l_: crown major axis; C_w_: crown minor axis.

## Data Availability

All data are presented in the main text.
